# Subfertility as Overlapping of Nutritional, Endocrine, Immune, and Cardiometabolic Dysregulations—A Study Focused on Biochemical Endophenotypes of Subfertile Couples

**DOI:** 10.3390/jcm12186094

**Published:** 2023-09-21

**Authors:** Tadeusz Wasilewski, Jolanta Wasilewska, Marta Łukaszewicz-Zając, Barbara Mroczko

**Affiliations:** 1 Centre for Restorative Procreative Medicine, Napromedica, 15-741 Bialystok, Poland; wasilewski_tadeusz@wp.pl; 2 Centre for Paediatrics, Allergology, Psychodietetics, and Treatment of Children Diagnosed with Autism, IPM, 15-404 Bialystok, Poland; 3 Department of Biochemical Diagnostics, Medical University of Bialystok, 15-269 Bialystok, Poland; marta.lukaszewicz-zajac@umb.edu.pl; 4 Department of Neurodegeneration Diagnostics, Medical University of Bialystok, 15-269 Bialystok, Poland

**Keywords:** biochemical biomarkers, biochemical endophenotypes, cardiometabolic dysregulations, gut microbiota, hypersensitivity, subfertility, thyroid autoimmunity

## Abstract

Subfertility is a global health issue, and as many as 30% of cases are attributed to unexplained reasons. A hypercaloric, high-fat diet stimulates the expansion of pro-inflammatory gut microbiota with a consequent rise in circulating lipopolysaccharides. Adverse gut microbiota remodeling can exacerbate insulin resistance, while sex and thyroid hormones may influence the variability in gut microbiota. This cross-sectional study included 150 participants and was designed to determine a biochemical, nutritional-related pattern that may distinguish subfertile from fertile individuals and couples. A panel of 28 biomarkers was assessed. Four biochemical phenotypes of unexplained subfertility were found, including two metabolic and two immune, when assessed using binary logistic regression models. Two phenotypes were distinguished in women: cardio-metabolic with atherogenic dyslipidemia (_Low_HDL-cholesterol: OR = 10.9; *p* < 0.05) and autoimmune thyroid disorder (_High_anti-thyroid-peroxidase: OR = 5.5; *p* < 0.05) and two in men: hepato-metabolic with elevated liver injury enzymes (_High_HOMA-IR: OR = 6.1; *p* < 0.05) and immune type-2 response (_High_IgE: OR = 6.4; *p* < 0.05). The chances of a couple’s subfertility rose with the number of laboratory components of metabolic syndrome in the couple (OR = 1.7; *p* < 0.05) and if at least one partner had an elevated total IgE level (>100 kU/L) (OR = 6.5; *p* < 0.05). This study found that unexplained subfertility may be accompanied by mutually overlapping immune and metabolic dysregulations in individuals and couples. We propose one-time laboratory diagnostics taking into account the lipid profile, insulin resistance, anti-thyroid-peroxidase, and total IgE in both males and females with unexplained subfertility. This may allow for a one-time assessment of targeted medical and nutritional interventions and help optimize patients’ health. The gut–organ axes related to subfertility are discussed in the context of the obtained results.

## 1. Introduction

Subfertility generally describes any form of reduced fertility with a prolonged time of unwanted non-conception [1]. Infertility is defined by the Practice Committee of The American Society for Reproductive Medicine (2019) as the failure to achieve a successful pregnancy after 12 or more months of regular, unprotected sexual intercourse. When the woman is aged 35 years or more, the time cut-off used to define subfertility is usually 6 months rather than 1 year [2]. This disorder may affect up to 186 million people worldwide and affects one in six couples, half of whom lack an explanation for their delay in conceiving [3,4].

Subfertility may be caused by factors concerning the male or female or both partners. It was estimated that 30% of infertility cases are attributed to male-only factors, while about 30% arise from problems concerning both partners [4,5,6]. Numerous factors have been reported to contribute to subfertility [7,8,9]. Some factors such as disorders of ovulation, including polycystic ovary syndrome, premature ovarian insufficiency, hypothalamic dysfunction, endometriosis, and cervical causes solely contribute to female infertility. There are several causes that are responsible for male infertility including disturbed sperm function, hypogonadism, blockages preventing sperm delivery, hormone imbalances, varicocele, and antisperm antibodies as well as malignancies and infections [7]. A growing body of evidence indicates that male-only factors contribute to approximately half of infertility cases with non-obstructive azoospermia being the most common [10]. Moreover, 8–28% of infertility cases remain unexplained, defined as having a lack of an obvious cause for a couple’s infertility [11].

Unexplained subfertility is relevant in reproductive medicine. Tests performed in accordance with current diagnostic protocols do not provide the answer(s) as to the cause of conception failure and pregnancy maintenance. Certain clinical investigations have reported that differences in biochemical parameters reflect endocrine, nutritional, immune, and cardiometabolic dysregulations in subfertile and fertile couples [12,13,14,15,16,17,18,19]. Recent studies indicate a potential relationship between fertility and nutrition and the gut microbiome. The quality of provided nutrients determines gut-related immune and metabolic health. A hypercaloric, high-fat diet may stimulate the expansion of a pro-inflammatory gut microbiota with a consequent rise of circulating lipopolysaccharides due to increased intestinal permeability [15]. The gut microbiota is the largest endocrine organ of the body and is constantly in a dynamic balance between eubiosis and dysbiosis. It has been proposed that sex hormones serve as major regulators of the gut microbiota’s variability [20,21,22]. Adverse gut microbiota remodeling can exacerbate insulin resistance. Higher fT4 concentrations have also been linked to subfertility with causes including environmental exposure to plastics and the metabolic effect of the gut microbiota [23]. Based on our knowledge, there are currently no original studies assessing the most crucial biochemical parameters reflecting these issues in infertile couples in comparison with healthy subjects with consideration of gender-specific ailments. This study was designed to determine a biochemical pattern that may distinguish subfertile from fertile individuals based on a total of 28 biomarkers of endocrine, immune, inflammatory, and metabolic status. The primary objective of this study was to determine a pattern in biochemical, nutrition-related indices of subfertile individuals in comparison with fertile subjects. An assessment of laboratory indices in subfertile vs. fertile couples was evaluated as secondary outcomes. The gut–organ axes related to subfertility are discussed in the context of the obtained results.

## 2. Materials and Methods

### 2.1. Patients and Samples

**Figure 1:**
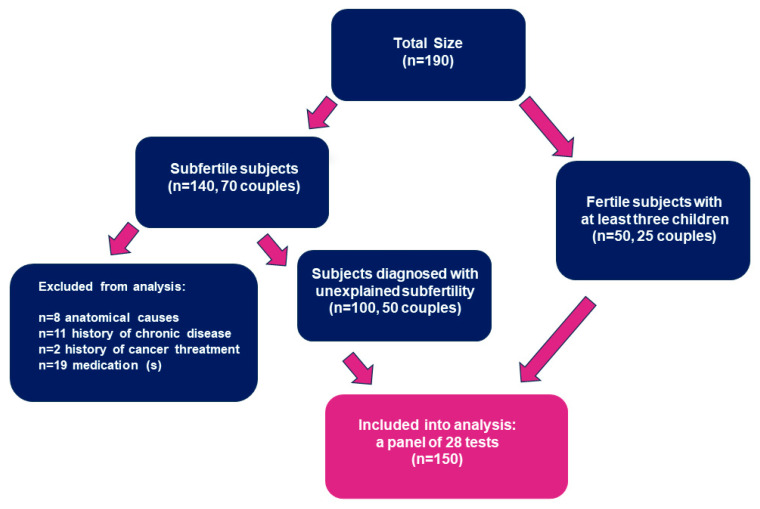
Flowchart showing the inclusion process used in this study.

The study group consisted of 100 patients (50 couples) suffering from infertility and diagnosed in the Centre for Restorative Procreative Medicine, Napromedica, Bialystok, Poland, between 28 September 2011 and 31 December 2012. The control group included 50 healthy volunteers (25 couples) who were recruited from hospital volunteer organizations and were parents of at least 3 children. Participants were included after providing informed consent. Inclusion criteria included patients who were diagnosed with unexplained subfertility, aged 25–50 years, without a history of chronic diseases (inflammatory bowel disease, Coeliac disease, rheumatoid arthritis, cancer), and were free of medications during recruitment for the study (before blood sampling). Exclusion criteria included patients who were aged under 25 and over 50 years, were diagnosed with sterility and anatomical causes of infertility, and did not provide informed consent. A flowchart of the study protocol is shown in Figure 1. Blood samples were acquired from patients in the morning after an overnight fast of ≥10 h and stored at −80 °C until analysis (Sarstedt, Nümbrecht, Germany).

### 2.2. Methods

All parameters were assessed using an ARCHITECT 8200 ci analyzer (Abbott, Abbott Park, IL, USA) and a COBAS E411 analyzer (ROCHE, Basel, Switzerland). Concentrations of C-reactive protein (CRP) and immunoglobulin A (IgA) were measured using the turbidimetric method. Serum levels of insulin, anti-thyroglobulin antibodies (anti-TG), anti-thyroid peroxidase antibodies (anti-TPO), and thyroid-stimulating hormone (TSH) were measured using the chemiluminescent microparticle immunoassay (CMIA) method. Alanine aminotransferase (ALT) and aspartate aminotransferase (AST) activity levels were assessed using the kinetic method, while serum levels of glucose were assessed using the hexokinase/G-6-PDH method. The concentrations of iron (Fe) were evaluated using the spectrophotometric method, whereas the photometric method was used to measure unsaturated iron-binding capacity (UIBC), sodium (Na), potassium (K), and the lipid profile: (triglyceride (–TGs), total cholesterol (T-CH), low-density lipoprotein cholesterol (–LDL-C), and high-density lipoprotein cholesterol (–HDL-C) concentrations). Sex hormone-binding globulin (SHBG) was assessed using an electrochemiluminescence immunoassay ECLIA with an Elecsys COBAS E411 analyzer (ROCHE, Basel, Switzerland). Serum levels of ImmunoCap total IgE and selected ImmunoCap specific IgE (sIgE) antibodies such as ImmunoCap Allergen f13 (peanut, Arachis hypogaea), ImmunoCap Allergen g6 (timothy, Phleum pretense), ImmunoCap Allergen o70 (Seminal fluid), ImmunoCap Allergen t3 (common silver birch, Betula verrucose), and ImmunoCap Allergen w6 (mugwort, Artemisia vulgaris) were measured using an fluoroenzymeimmunoassay (FEIA) with a Phadia 100 immunoassay analyzer (Thermo Fischer, Waltham, MA, USA).

### 2.3. Laboratory Criteria

#### 2.3.1. Thyroid Function Tests

The frequency distribution of TSH was compared in both groups in the following ranges: TSH < 1.5, TSH1.5 < 2.5, and TSH ≥ 2.5 µIU/m [24]. Levels of anti-TG > 4.1100 IU/mL and anti-TPO > 5.6100 IU/mL were defined as antibody-positive according to the manufacturer’s instructions (Abbott, Abbott Park, IL, USA).

#### 2.3.2. Lipid, Liver, and Carbohydrate Metabolism

Laboratory criteria for metabolic syndrome were selected according to the National Cholesterol Education Program Adult Treatment Panel III (NCEP ATP III): blood triglycerides > 150 mg/dL (>1.7 mmol/L); HDL-cholesterol < 40 mmol/L (<1.0 mmol/L) for males; and HDL-cholesterol < 50 mg/dL (<1.3 mmol/L) for females [25]. The Castelli Risk Index I (CRI-I), an atherogenicity index, evaluates the ratio of the atherogenic lipid component (TC) to the anti-atherogenic (HDL-C) component, and a value of <4 was considered low risk in the lipid profile [26]. The optimal value of LDL-cholesterol levels for the general population, as recommended by the European Society of Cardiology and European Atherosclerosis Society (ESC/EAS), is 116 mg/dL [27]. ALT activity above 19 IU in women and above 30 IU in men was considered as over optimal values according to the American College of Gastroenterology Clinical Guidelines: Evaluation of Abnormal Liver Chemistries [28]. Insulin resistance (IR) values were estimated using the homeostasis model assessment (HOMA-IR). HOMA-IR was calculated using the mathematical equation from Matthews et al.: insulin (μU/mL) × glucose (mmol/L)/22.5 [29]. The HOMA-IR ratio was used as a measure of insulin resistance with a cut-off level of 1.9 [30].

#### 2.3.3. Total IgE and Allergen-Specific IgE Sensitization

The results for sIgE concentrations are reported as a class system (classes 0–6) based on the amount of detected serum-specific IgE. The ImmunoCAP-specific IgE classes are defined using six calibrators: 0, 0.35, 0.7, 3.5, 17.5, and 100 kU_A_/L (class 0: from 0 to <0.35 kU_A_/L; class 1: from 0.35 to <0.7 kU_A_/L; class 2: from 0.70 to <3.5 kU_A_/L; class 3: from 3.50 to <17.5 kU_A_/L; class 4: from 17.5 to <50 kU_A_/L; class 5: from 50 to <100 kU_A_/L; and class 6: from ≥100 kU_A_/L), where kU_A_/L: kilounits of allergen-specific IgE per liter [31]. Higher sIgE concentrations are associated with a greater likelihood that a patient may suffer from allergic symptoms caused by exposure to the sensitizing allergen. The lower detection threshold for sIgE determination is 0.35 kU_A_/L. The presence of sIgE against a particular allergen above this level is interpreted as positive for that allergen, and a positive test for aeroallergens generally correlates well with the clinical expression [32].

### 2.4. Statistical Analysis

Variables were checked for normal distributions before statistical analyses using the D’Agostino–Pearson normality test (the “omnibus K2”) (GraphPadPrism5, SanDiego, CA, USA, www.graphpad.com, (accessed on 6 March 2023)). Quantitative data and non-normally distributed variables were expressed as the median (second quartile, Q2) and interquartile range (IQR). Normally distributed variables were expressed as the mean ± SEM and 95% confidence interval (CI). Qualitative data were expressed as frequency and percentages. Data were compared with a non-parametric test using the Wilcoxon rank-sum two-tailed test (Mann–Whitney *U*). Proportions between categorical variables were compared using a two-tailed Fisher’s exact test. Spearman’s rank correlation coefficient (R_s_) was used to measure the strength and direction of associations between inflammatory and metabolic indices: C-reactive protein vs. lipid profile indices and insulin resistance indices. Binary logistic regression models were used to analyze laboratory predictors of subfertility and to calculate odds ratios (ORs) with 95% CIs (confidence intervals). A receiver operating characteristic (ROC) curve analysis was used to calculate the cut-off value for serum levels of free T4 in subfertile subjects. Probability (*p*) values less than 0.05 were considered significant.

## 3. Results

**Figure 2:**
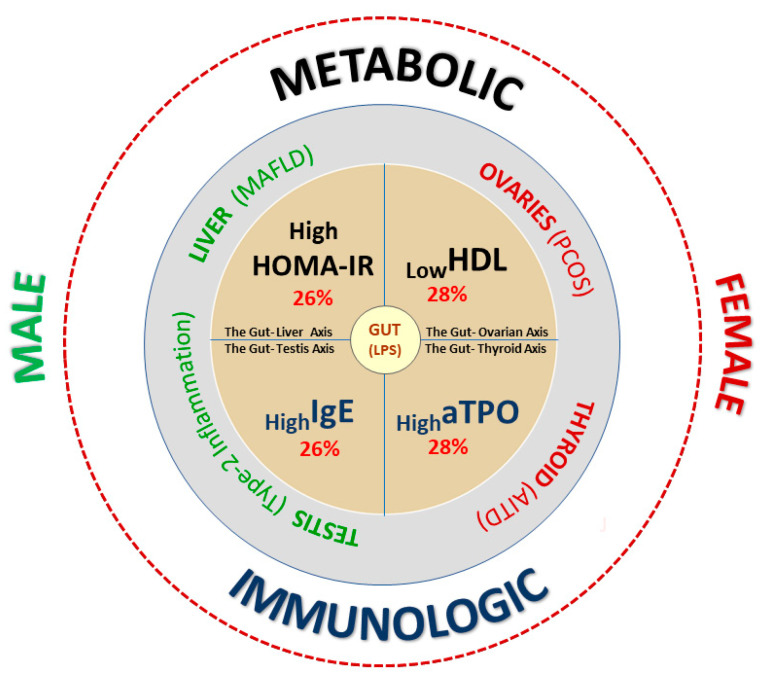
An infographic summary of the study results. Four biochemical endophenotypes of subfertile individuals were found: two metabolic and two immune, including two in women and two in men. In women: “the cardio-metabolic _Low_HDL” and “the autoimmune _High_anti-TPO”. In men: “the hepato-metabolic _High_HOMA-IR” and “the immune type-2 response _High_IgE”. The target organs potentially disorganized in these phenotypes were: the ovaries and thyroid in women and the liver and testes in men. The blood analysis showed subfertility accompanied by atherogenic dyslipidemia, insulin resistance, and an increased immune humoral response: autoimmune and type-2 inflammation. These results reflect systemic low-grade inflammation with immune and metabolic interplay (“subfertile infla-metabolom”), which, to a certain extent, may be a result of intestinal microbial stimulation by lipopolysaccharides (LPSs). Four microbiota–gut–organ axes are potentially involved in these results: the gut–ovaries axis (GOA), the gut–thyroid axis (GThA), the gut-liver axis (GLA), and the gut-testis axis (GTA).

This study included one hundred patients diagnosed with unexplained subfertility and fifty parents had at least three children. The median age was 32 years (IQR: 29–34) (min–max 25–41) in the group of subfertile women and 33 years (IQR: 30–36) (min–max 29–45) in the fertile women (*p* > 0.5). The median age of subfertile men was 34 years (IQR: 30–36) (min–max 25–48) and 34 years (IQR: 31–39) (min–max 29–50) for fertile men (*p* > 0.5). The main biochemical features of the subfertile men and women are summarized in Figure 2.

### 3.1. Thyroid Function and Thyroid Autoimmunity

#### 3.1.1. Thyroid Function Tests: Thyroid-Stimulating Hormone (TSH) and Free Thyroxine (fT4)

**Figure 3:**
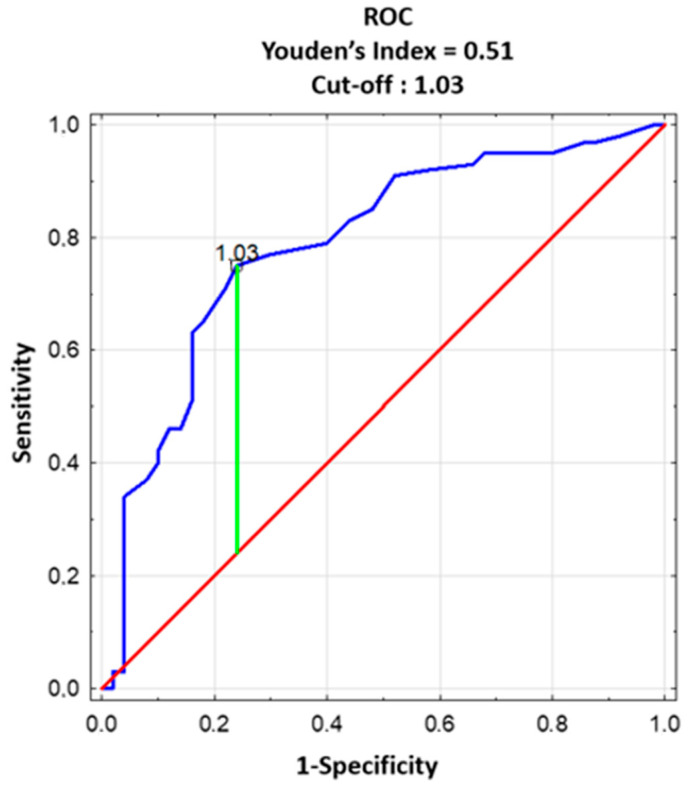
The receiver operating characteristic curve (ROC) analysis for the prediction of subfertility based on the serum level of free T4. The cut-off level for free T4 value = 1.03 for subfertile subjects, AUC: area under the ROC curve = 0.787, with a diagnostic sensitivity of 75% and diagnostic specificity of 76%. Blue line: ROC curve; Red line: Chance level (square diagonal); Green line: maximum value of Youden’s index for the ROC curve. Youden’s Index: J = sensitivity + specificity − 1. The index is defined for all points of an ROC curve, and the maximum value of the index may be used as a criterion for selecting the optimum cut-off point when a diagnostic test gives a numeric rather than a dichotomous result. The index is represented graphically as the height above the chance line, and it is also equivalent to the area under the curve subtended by a single operating point.

TSH concentration was comparable in both groups as well as the frequency distribution of TSH. In almost 60% of both groups, the TSH concentration was below 1.5 µIU/mL (Table S1). Serum fT4 concentration was slightly and statistically significantly higher in the subfertile group than in the fertile group (Table S1). In the ROC analysis, the cut-off value of fT4 for subfertile subjects was 1.03 ng/dL with a maximum Youden index of 0.51, area under the curve (AUC) of 0.787, accuracy of 0.753, diagnostic sensitivity of 75%, diagnostic specificity of 76%, positive predictive value (PPV) of 86%, and negative predictive value (NPV) of 60% (Figure 3).

#### 3.1.2. Autoimmune Thyroid Disorder (AITD): Anti-Thyroid Peroxidase Autoantibodies (Anti-TPO) and Anti-Thyroglobulin Autoantibodies (Anti-TG)

Anti-TPO positive sera were found more often in subfertile than fertile participants, in 22% vs. 8%, respectively (Table S1). Serum anti-TPO antibodies correlated positively with TSH (R_s_ = 0.377, *p* < 0.001) and with HDL-C (R_s_ = 0.313, *p* < 0.01) in the subfertile group but not in the fertile group. There was a positive correlation between serum anti-TG autoantibodies and TSH (R_s_ = 0.35, *p* < 0.001) in the subfertile group but not in the fertile group.

#### 3.1.3. Biochemical Characteristics of TPO-Positive Subfertile Participants

**Figure 4:**
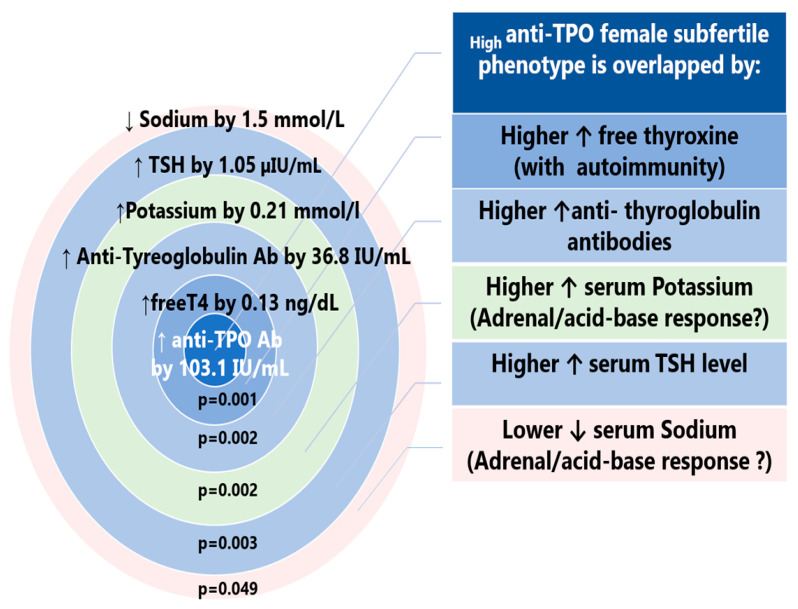
The autoimmune _high_anti-TPO female subfertile endophenotype: a comparison of blood analyses to the fertile, anti-TPO-negative women (TPO—thyroid peroxidase). Associated differences in blood biochemical indices: higher serum free T4, higher serum anti-thyroglobulin antibodies, higher potassium concentration, higher TSH level, and lower serum sodium concentration. ↑—higher; ↓—lower; Ab—antibodies.

TPO-positivity in the subfertile group was associated with differences in certain biochemical results (Table S2). Subfertile TPO-positive subjects differed from the TPO-negative fertile subjects in thyroid function tests, in TSH, fT4, and electrolytes (Na, K) (Table 1, Figure 4).

### 3.2. Lipid Profile

**Figure 5:**
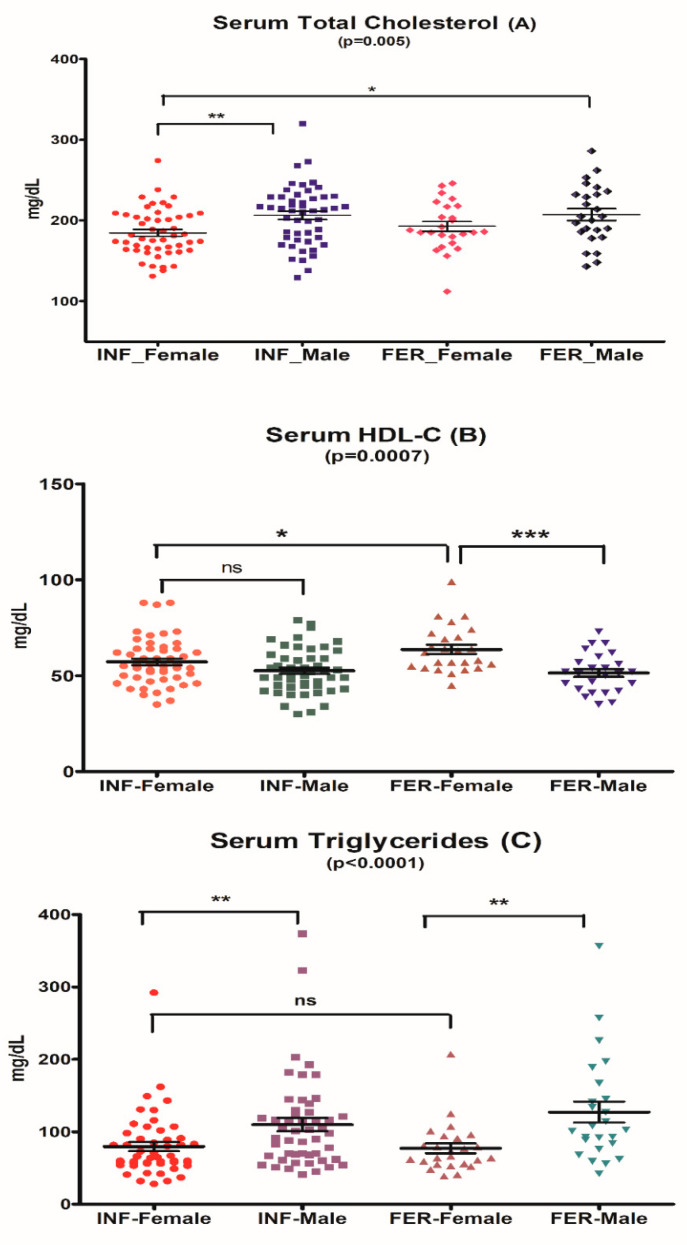
Lipid profile characteristics of subfertile (INF) and fertile (FER) males and females. * *p*-value < 0.05; ** *p*-value < 0.01; *** *p*-value < 0.001.

The characteristics of lipid profile measurements in the study participants are presented in Table S2 and Figure 5.

#### 3.2.1. Serum Total Cholesterol

The intra-group analysis showed significant gender-related differences in serum total cholesterol (Figure 5A). The lowest total cholesterol level was found in subfertile women and was significantly lower than in subfertile men (185 mg/dL [95% CI 176.4–193.0 mg/dL] vs. 206 mg/dL [95% CI 195.8–216.7 mg/dL], respectively, *p* = 0.0013). Fertile women did not differ from fertile men in total serum cholesterol.

#### 3.2.2. Serum LDL-Cholesterol

No differences were observed in serum LDL-cholesterol between subfertile and fertile groups, between subfertile and fertile women, or between subfertile and fertile men (Table S2). In the subgroup of subfertile men with higher than optimal LDL-C above 120 mg/mL, liver indices were found to be elevated (higher insulin resistance and liver enzyme activity) (Table S2).

#### 3.2.3. Serum Triglycerides

The differences in serum TG levels were sex-related. Both subfertile and fertile women differed from men with lower triglyceride levels (*p* = 0.003 and *p* = 0.002, respectively). No differences were observed in serum TG between the subfertile and fertile groups (Figure 5C).

#### 3.2.4. Serum HDL-Cholesterol

**Figure 6:**
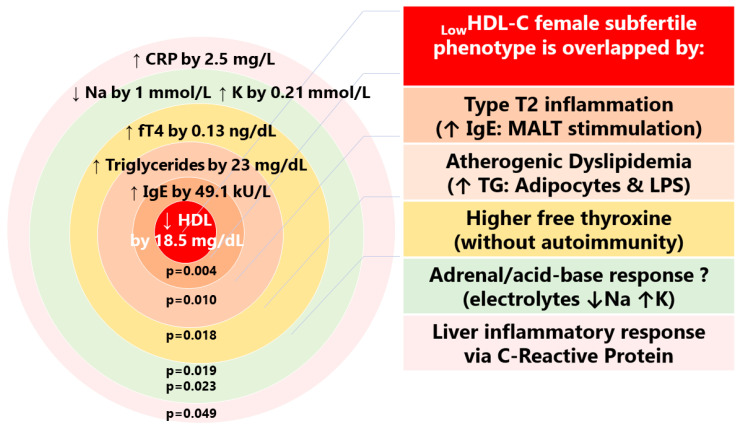
The cardio-metabolic _Low_HDL female subfertile endophenotype: a comparison of blood analyses of fertile women with HDL > 50 mg/dL. Associated differences in blood biochemical indices: higher serum total IgE, higher serum triglycerides, higher free T4, lower sodium and higher potassium concentrations, and higher C-reactive protein. ↑—higher; ↓—lower.

Low serum HDL-C < 50 mg/dL was observed more often in subfertile than in fertile women, in 28% vs. 4.0% (Table S2, Figure 5B). The low HDL-C female phenotype was associated with differences in blood biochemical indices: higher serum total IgE, higher serum TG, higher free T4, lower sodium and higher potassium, and higher CRP levels (Table 2, Figure 6).

#### 3.2.5. Spearman’s Correlations for Lipid Profile Indices

Lipid profile indices significantly correlated with CRP exclusively in the subfertile group (Table 3).

### 3.3. Glucose and Insulin Levels and HOMA-IR

Elevated fasting insulin ≥ 12 uU/mL was noted in 13% of the subfertile group and in 2% of the fertile group (Table S3. The HOMA-IR from the fasting plasma glucose and insulin concentrations were significantly higher in the subgroup of subfertile men with LDL-C above 120 mg/dL (Table S2). Biochemistries of the subfertile _High_HOMA-IR male phenotype differed from controls in liver markers: higher liver enzyme alanine transaminase (ALT) activity, a higher proportion of men with ALT > 30 IU, indicating liver cell injury, and lower SHBG levels (Table 4).

### 3.4. Total IgE and Allergen-Specific IgE Sensitization

Total serum IgE was higher in the subfertile than fertile group (Table S3). In the subfertile group, 13 out of 21 (62%) participants with total IgE above the upper reference limit (URL) were men and 70% of them were non-atopic (Table 5).

#### sIgE Sensitization

Seminal plasma hypersensitivity (SPH) was excluded in all subfertile and fertile participants based on a lack of serum seminal fluid sIgE antibodies. Allergen-specific IgE sensitization to food or to pollen allergens was found in 13 out of 100 subfertile persons and in 3 out of 50 fertile persons (*p* = 0.654). Total IgE was comparable in these subjects: 106 kU/mL vs. 86.3 kU/mL (*p* = 0.346), respectively. Food sensitization represented by peanut sensitization (sIgE f13, class 2) was diagnosed in three subfertile subjects, and in all these persons, it was accompanied by sensitization to grass and weeds. All the peanut-sensitized subjects were free of thyroid autoimmunity. Grass pollen sensitization (sIgE g6, class 2–5) was found in 11 subfertile (M:F = 7:4) and 2 fertile participants (M:F = 1:1) (*p* = 0.221). Tree pollen sensitization was found in men only in three subfertile and two fertile subjects. Weed sensitization (sIgE w6) was observed only in the subfertile group (four subjects, M:F = 2:2). Weed sensitization coexisted with peanut and grass sensitization as a polyvalent sensitization in three out of four persons. All these pollen-sensitized subjects were free of thyroid autoimmunity.

### 3.5. Laboratory Test Result Comparison between Couples

**Figure 7:**
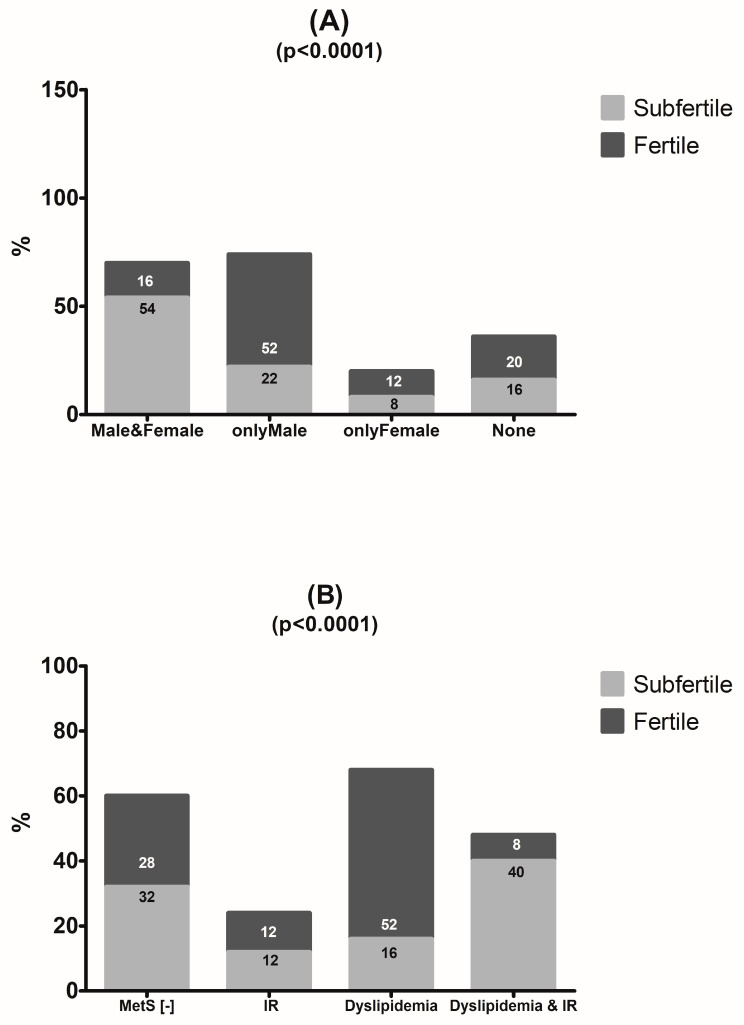
Analysis of the frequency distribution of positive laboratory results in couples. (**A**) Components of metabolic syndrome, TPO positivity, and/or total IgE above the URL were found in 54% of subfertile couples both in men and women vs. 16% of fertile couples. In 52% of fertile couples, positive laboratory results were found only in men. (**B**) Frequency distribution of biochemical components of metabolic syndrome in subfertile and fertile couples. Both atherogenic dyslipidemia and insulin resistance were found in 40% of subfertile couples, while 52% of fertile couples showed isolated, single features of dyslipidemia.

**Figure 8:**
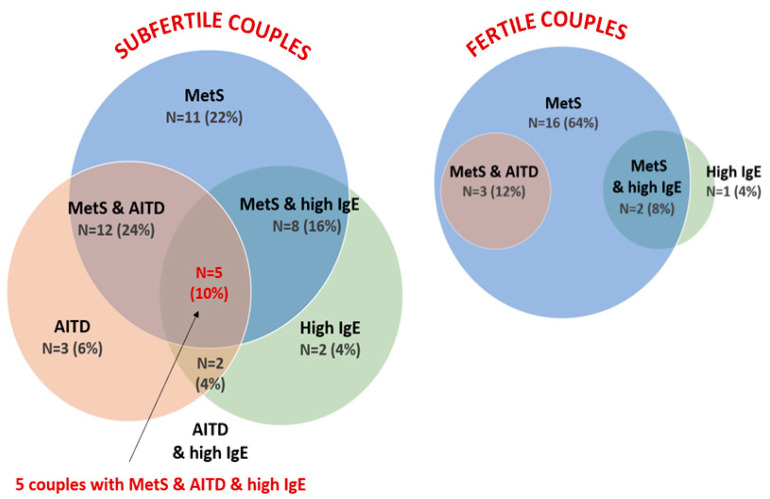
The overlapping of laboratory components of metabolic syndrome (MetS) by autoimmune thyroid disorder (AITD) and/or increased total serum IgE above the URL (_High_IgE) in subfertile and fertile couples.

Components of metabolic syndrome, TPO positivity, and/or total IgE above the URL were found in 54% of subfertile couples, both in men and women, vs. 16% of fertile couples. In 52% of fertile couples, positive laboratory results were found only in men (Figure 7A). An analysis of frequency the distribution of biochemical components of metabolic syndrome in subfertile and fertile couples showed that 40% of subfertile couples had both atherogenic dyslipidemia and insulin resistance, while 52% of fertile couples showed isolated, single features of dyslipidemia (Figure 7B). In 20 out of 50 (40%) subfertile couples, components of metabolic syndrome (MetS) were found both in the males and females as compared with 3 out of 25 fertile couples (12%) (*p* = 0.017) (Figure S1). The overlapping of laboratory components for MetS, AITD, and increased total IgE above the URL (_High_IgE) was found in 50% (25/50) of subfertile vs. 20% (5/25) of fertile couples (Figure 8).

In 20 out of 50 (40%) subfertile couples, components of MetS were found both in the males and females as compared with 3 out of 25 fertile couples (12%) (*p* = 0.017; Fisher’s exact test; two-sided *p*-value).

### 3.6. Laboratory Predictors of Individual and Couple Subfertility

The analysis indicated an overlap between immune and metabolic dysregulation in subfertile individuals and couples, which increased the chance of subfertility in the couple. Significant predictors of male subfertility were HOMA-IR > 1.9 and serum IgE above 100 kU/L (Table 6A). Significant predictors of female subfertility were HDL-C < 50 mg/dL and anti-TPO positivity (Table 6B). The chances of a couple’s subfertility rose with the number of laboratory components of metabolic syndrome in the couple and if at least one partner had an elevated total IgE level (>100 kU/L) (Table 6C).

## 4. Discussion

The present study found two significant metabolic (_Low_HDL-C and _High_HOMA-IR) and two immune (_High_anti-TPO and _High_IgE) laboratory predictors of individual subfertility. Specific organ disorganization can be assigned to these biochemical subtypes. For the female cardio-metabolic _Low_HDL endophenotype, the potentially disorganized target organs are the ovaries, represented by PCOS. For the autoimmune _High_anti-TPO female endophenotypes, the target organ is the thyroid, represented by AITD. For the male hepato-metabolic _High_HOMA-IR endophenotype, the potential target organ is the liver, represented by MAFLD. For the _High_IgE male endophenotype, the potential target organ are the testes through type-2 inflammation involving stimulation of MALT and GALT (mucosa–gut-associated lymphoid tissue) and thereby influencing testicular function via the gut-testes axis. These four phenotypes illustrate four functional inter-organ axes important for human fertility: the gut–ovarian axis, the gut–thyroid axis, the gut–liver axis, and the gut–testes axis, respectively. The gut is a common, connecting element of these axes.

### 4.1. Cardio-Metabolic Female Phenotype of Subfertility (_Low_HDL-Cholesterol)

This study found that women in the subfertile group had an HDL-C concentration that was 6.5 mg/dL lower than women in the fertile group. This result is similar to that described in women with PCOS in the Wild at al. meta-analysis, where a value of 6 mg/dL was found [33]. PCOS, a systemic endocrine disorder, is associated with adipose tissue dysfunction and an increased risk of insulin resistance, metabolic dysfunction-associated fatty liver disease (MAFLD), and dyslipidemia [4]. Lipid alterations in PCOS mainly include a decrease in HDL-C and an increase in TG [34]. This study found that 28% of subfertile women vs. 4% of fertile women had an HDL-C below a lower range of 50 mg/dL (_Low_HDL-C phenotype). In the general population, PCOS is diagnosed in up to 20% of women using an ultrasound scan, and around 7% of women have additional features of PCOS [35]. In the subset of subfertile women with a _Low_HDL-C phenotype, the serum HDL-C level was lower by 18.5 mg/dL than in the control fertile women.

In observational epidemiology, the inverse association between plasma HDL-C and the risk of atherosclerotic cardiovascular disease is among the most consistent associations. However, the latest research reveals new evidence for the role of HDL-C in female fertility: HDLs help in the implantation and development of the embryo and contributes to a proper course of pregnancy [27,36,37,38]. Several mechanisms underlying low HDL-C may be involved in female infertility and be related to: (i) an ovulatory dysfunction, (ii) an inflammatory response involving the reproductive organ, and/or (iii) an epigenetic/genetic background of decreased fertility. Referring to the first issue, HDLs are the only lipoproteins detected in substantial amounts in follicular fluid. Their size and composition correlate with embryo quality [39]. In contrast with low serum concentrations of HDL cholesterol, high HDL-C levels have been associated with better oocyte and embryo outcomes as well as having beneficial effects on spermatogenesis [40]. Referring to the second issue, it has been shown that a decrease in HDL-C may be induced by increased expression of pro-inflammatory cytokines, i.e., those of the tumor necrosis factor-alpha (TNF-α) family [41,42]. Therefore, inflammatory diseases and an altered reproductive tract microbiome (as seen in endometriosis, pelvic inflammatory disease, or obesity-induced inflammation) and gut microbiota disturbances with many pro-inflammatory cytokines are taken into account as cofactors of ovary dysfunction [43,44,45]. With regard to the third issue, epigenetic studies have revealed that low HDL-C was the only metabolic marker significantly associated with epigenetic age acceleration (EAA) in mothers [46]. This means that the _Low_HDL cardio-metabolic phenotype of subfertility can be considered the first sign of an “accelerated aging disease” [47]. Ovaries could be the first compartment to show signs of a general process of accelerated aging, and infertility could be considered part of a more general systemic illness that is defined by accelerated or premature aging processes [48].

The second component of lipid dysregulation in the subset of women with a _Low_HDL-C phenotype was higher serum triglyceride levels by 23 mg/dL as compared with the control group of fertile women. This result is similar to that described in women with PCOS in the Wild at al. meta-analysis, where a value of 26 mg/dL was observed [33]. A lipid profile based on decreased HDL-C and higher TG is a phenotype reflecting insulin resistance [42]. Reduced high-density lipoprotein cholesterol and raised triglycerides were found as the main individual components associated with risk for infertility in the multicenter study by Grieger et al. that included 5519 women [49]. An increased synthesis and secretion of triglyceride-rich lipoproteins by the liver was described as the host’s response to infection—a reaction to the bacterial endotoxins such as lipopolysaccharides (LPSs) and gut dysbiosis [50,51]. LPS may also play a role in inducing ovarian dysfunction via hypothalamic LPS receptors and may have an inhibitory effect on pulsatile GnRH/LH release regulated by kisspeptin signaling [52].

As additional support for metabolic dysregulation in the subset of _Low_HDL-C subfertile women, a significantly lower level of circulating SHBG was found compared with subfertile women with normal levels of HDL-C (53.1 vs. 71.2 nmol/L; *p* = 0.016). A reduction in the SHBG concentration, a globulin produced mainly in the liver, is associated with PCOS and is a marker of the severity of hepatic insulin resistance. The expression of SHBG mRNA was found to be negatively correlated with the accumulation of triglycerides in hepatocytes [53]. ALT, a marker of liver cell injury, was elevated above 19 IU/mL in 35.7% of _Low_HDL-C subfertile women as compared with 4% of fertile women with a level of HDL-C above 50 mg/mL.

Furthermore, the _Low_HDL-C female phenotype in this study was associated with significantly higher serum total IgE by 49.1 kU/L, representing type-2 inflammation. The significance of this observation is not clear, and it is not known whether it is related to the pathomechanisms of infertility. It would be prudent to keep this in mind in view of new discoveries in uterine and vaginal mucous immunology [54]. Vaginal tissue-resident lymphocytes are able to produce a vaginal T_H_1 response (after infectious stimulation) and also induce vaginal T_H_2 humoral immunity with type-2 cytokines including interleukins 4, 5, and 13 (IL-4, IL-5, IL-13). Also, the human endometrium contains lymphoid aggregates that share organizational similarity to isolated lymphoid follicles (ILFs) and Peyer’s patches in the intestinal mucosa. However, their biological function remains unclear [54].

### 4.2. Hepato-Metabolic Male Phenotype of Subfertility (_High_HOMA-IR)

The hepato-metabolic male phenotype was identified as a subset in 26% of subfertile men with LDL-C > 120 mg/dL and HOMA-IR > 1.9 as compared with 8% of fertile men. This phenotype was characterized by higher levels of insulin, higher activity of alanine transaminase (ALT), a lower AST to ALT ratio (below 1), and a lower concentration of SHBG. These biochemical indices belong to laboratory markers of MAFLD in which hepatic insulin resistance and dysregulation of adiponectin play major roles [28,55]. Increased ALT, a specific marker of hepatocellular cell injury and fat accumulation in the liver, is responsible for a decline in the aspartate aminotransferase-to-alanine transaminase ratio (AST:ALT) [56]. Insulin disorders affect male infertility by decreasing spermatogenesis and semen parameters, reducing vacuolization in the Sertoli cells, and decreasing Leydig cell counts and testosterone levels [57,58]. Increased insulin resistance was described by Mansour et al. in 160 men with unexplained infertility having concomitant increases in FSH and LH and a decreased level of testosterone [59]. Increased insulin is known to inhibit SHBG synthesis. Serum levels of SHBG measured in this study were 31% lower in the group of men with insulin resistance. In a cohort of 1896 non-diabetic middle-aged Finnish men, Laaksonen and associates found that men with MetS had an 18% lower SHBG than controls [60]. Adverse gut microbiota remodeling can exacerbate insulin resistance via LPS-mediated modulation of toll-like receptor (TLR) signaling. A hypercaloric, high-fat diet can induce insulin resistance by stimulating the expansion of pro-inflammatory gut microbiota with a consequent rise in circulating LPS due to increased intestinal permeability [15]. Conversely, certain probiotic strains improve fasting glycemia, hyperinsulinemia, HOMA-IR, and glycated hemoglobin via gut peptide secretion. MAFLD reflects gut–liver axis dysregulation and is associated with impairment of the intestinal barrier (increased serum diamine oxidase) and translocation of gut bacterial metabolites (increased serum LPS and D-lactate) [61]. MAFLD is also characterized by increased production of secondary bile acid by taurine- and glycine-metabolizing bacteria in the gut. Increased levels of specific bile acids, such deoxycholic acid (DCA), have been negatively correlated with male fertility [21]. Bile acids regulate testosterone synthesis within Leydig cells through the nuclear receptor FXRα (Farnesoid-X-receptor alpha) and the G-protein-coupled bile acid receptor (GPBAR-1) resulting in decreases in testosterone levels and decreased fertility [62].

### 4.3. Autoimmune Female Phenotype of Subfertility (_High_Anti-Thyroid Peroxidase)

The autoimmune _High_anti-TPO female endophenotype was characterized by a significantly higher TSH level and higher serum concentration of fT4, although within the normal range, and dissociation of serum levels of electrolytes: higher potassium and lower sodium levels compared with the fertile women without autoimmunity. Anti-TPO autoantibodies were noted in 28% of subfertile vs. 8% of fertile women. In general, in the unselected population, the prevalence of antithyroid antibodies ranged from 6% to 20%, being higher in women with a history of subfertility or recurrent pregnancy loss, in whom the prevalence reached up to 31–33% [63]. Anti-TPO positivity is a marker of autoimmune thyroid disorders (Graves’ Disease and Hashimoto Thyroiditis) and is linked with male and female subfertility, even in subjects with biochemically normal thyroid function [64,65]. Thyroid autoimmunity has been mentioned in various possible pathogenetic mechanisms: premature ovarian aging with lower anti-Müllerian hormones, an increased risk of ovarian hyperstimulation, antiphospholipid antibody syndrome, cross-reactivity between anti-thyroid and anti-zona pellucida antibodies, endometriosis, genetic components, and systemic immune dysfunction with inhibition of immune tolerance [63,66,67]. A meta-analysis of four studies showed that among antibody-positive euthyroid women, the risk of unexplained infertility is increased (OR 1.5, 95% CI 1.1–2.0) [68]. In this study, serum levels of TSH were within the normal range but were higher by 1.05 µIU/mL in subfertile anti-TPO positive vs. fertile anti-TPO negative women. In a meta-analysis of six studies, women with thyroid autoimmunity had higher serum TSH by 0.51 mIU/L compared with a negative group [63]. Another analysis of 22 clinical studies had a higher level of TSH by 0.61 mIU/L (Wang, 2021 #549). Women in the subfertile group had higher serum concentrations of fT4, which accounted for nearly 90% of the normal range, as compared with 32% of the normal range in the group of fertile women. In recent years, extra-thyroid causes of higher fT4 concentrations have been described to be linked to subfertility and include environmental exposure to plastics or the metabolic effects of gut microbiota [23,69,70]. Thyroid homeostasis is a dynamic component of the gut–thyroid axis [71]. Parameters of thyroid function such as triiodothyronine (fT3), fT4, and anti-TPO showed the most significant positive and negative correlations between bacterial levels, suggesting that the thyroid is under the influence of the metabolic function of intestinal microorganisms [72]. The role of the microbiome in thyroid function results from the enterohepatic circulation of thyroid hormones, iodothyronine metabolism, and iodine and selenium intake status. Two important gut players in this interplay are the microbiota derivatives LPSs and short-chain fatty acids (SCFAs), which act in several ways [73]. LPSs directly affect thyroid cells by increasing the expression of TSH and NIS genes (the sodium/iodine symporter); LPSs mediate downstream activation of nuclear factor kappa-B (NF-kB) through toll-like receptor 4 (TLR-4) on thyroid cells to regulate thyroid cell function. LPSs can inhibit the activity of hepatic type I iodothyronine deiodinase (D1) and conversely activate type II iodothyronine deiodinase (D2) in the hypothalamus and anterior pituitary, thus facilitating the conversion of T4 into T3, which affects thyroid function [73]. Short-chain fatty acids, together with thyroid hormones, may promote enterocyte differentiation and strengthen intercellular tight junctions, the latter being crucial components of the intestinal barrier ensuring its integrity [74]. The role of the gut–thyroid axis in AITD is linked with molecular mimicry, where an infectious agent shares a protein epitope with a self-protein within the peripheral target tissue and enhances an autoimmune attack. The abundance of 18 types of gut bacteria (for example Helicobacter pylori, Yersinia enterocolitica, etc.), was demonstrated to be linked with infection-associated autoimmune thyroiditis and positively correlated with anti-TPO and anti-TG [74,75].

### 4.4. Type-2 Immune Response Phenotype of Subfertility (_high_IgE) in Men

Subfertile participants were found to have higher concentrations of total serum IgE than controls, particularly in two subgroups: in the subgroup of males with IgE > 100 kU/L (higher by 147.5 kU/L) and in the subgroup of subfertile low HDL females (higher by 49.1 kU/L). Markers of atopy, allergen-specific IgE, were similarly distributed in the subfertile and fertile groups, whereas subfertile _High_IgE men were mostly non-atopic. Increased IgE production is one of the hallmarks of a type-2 immune response. Type-2 inflammation is a specific type of immune response pattern in which group 2 innate lymphoid cells (ILC2s) and Th2 lymphocytes play a key role with type-2 cytokines (IL-4, IL-13, IL-5, IL-25, IL-31, and IL-33) [76]. A type-2 immune response protects against parasites and is involved in certain chronic medical conditions such as atopic dermatitis, chronic rhinosinusitis with nasal polyps, certain types of asthma, alopecia, and eosinophilic esophagitis. Production of IgE in a type-2 response is induced by many non-infectious environmental antigens (food components, pollen, toxins) and infectious antigens (parasite infections such as tapeworms, nematodes, protozoa, and viral infections and infections with toxin-producing bacterial species such as *S. aureus*) [77]. This study found no differences in food and pollen sIgE sensitization in the subfertile vs. fertile groups. Ekladios et al. described a significantly higher serum IgE in a subgroup of men with obstructive azoospermia, especially when associated with an infection [78]. Microbial infections; viruses such as Epstein–Barr virus (EBV), cytomegalovirus (CMV), *Herpes* viruses, and hepatitis viruses; fungi; and bacteria (*Mycoplasma*, *Chlamydia*, *Ureaplasma*, *Neisseria*) are common causes of male infertility, possibly resulting in varicoceles, orchitis, prostatitis, oligozoospermia, asthenospermia, and azoospermia [79]. An occurrence of raised IgE levels was observed significantly more often in infertile couples than in a comparison group, as described by Harrison and Unwin [80]. In opposition to this study’s result, Hanzlikova et al. described a lower frequency of elevated total and allergen-specific IgE in women with reproduction failure compared with controls [81]. An increased IgE response was described in subjects with significantly elevated antisperm antibody titers [82], but the described seminal plasma hypersensitivity was not found in any of this study’s participants. A critical mediator of an IgE-mediated reaction is histamine, synthesized from the amino acid histidine through the enzyme histidine decarboxylase (HDC) [83]. In Houle et al.’s metabolomic study, histidine metabolism was associated with the differentiation between low sperm quality and normal sperm quality [84]. The main effector cells for IgE are mast cells, basophils, eosinophils, and histaminergic neurons. Human neutrophils are also able to produce and release histamine after antigenic activation through IgE receptors with high affinity (FcƐ RI) as those with low affinity (FcƐRII/CD23) [85]. Matalliotakis et al. found increased levels of soluble CD23 in subfertile men with abnormal spermiograms compared with men with normal sperm quality, suggesting that men with idiopathic testicular lesions may be immunologically more active than other groups with subfertility [86]. It has been shown that histamine can be released from neutrophils not only after allergen stimulation but also after bacterial and endotoxin stimulation. Additionally, antibiotics can reduce the production and concentration of IgE [85,87]. In the gut–testis axis, the diversity of the gut microbiome plays a key role that further influences the testicular microbiota. The translocation of gut bacteria and fecal metabolites (LPS) into the systemic circulation and then crossing the blood–testis barrier (BTB) may impact androgen production, and spermatogenesis, and contribute to reduced fertility [88]. The BTB includes tight junctions, gap junctions, desmosome-like junctions, and Sertoli cells. Histamine appears to increase permeability, acting through the actin cytoskeleton to induce physical gaps between these cells and components and thereby contributes to the increased permeability in the blood–testis barrier [89]. The role of IgE, a predictor of male subfertility in this study, and its mediator histamine may be supposed; however, whether and how high IgE is causally related to infertility requires further research.

### 4.5. Metabolic Predictors of Couple Subfertility

Laboratory metabolic predictors of subfertility evaluated in this study, both low HDL-C in women and high HOMA-IR in men, belong to biochemical components of metabolic syndrome. The odds of infertility in couples increased with the number of analyzed biochemical components of MetS. The metabolic-inflammatory component shared with subfertility was recently described in infertile males and females [14,39]. A growing number of recent human studies suggest the role of lipids in maintaining female and male fertility [40,84].

MetS, a pro-inflammatory state, is characterized by increased inflammatory cytokine activity and represents a state of metabolic inflammation (metaflammation) [90,91,92]. In the entire group of subfertile individuals, a significant correlation between C-reactive protein and the biochemical components of metabolic syndrome was observed. Additionally, recent studies show that metabolic dysfunction and low-grade chronic inflammation can trigger and enhance each other and that certain pro-inflammatory cytokines have the capacity to induce dyslipoproteinemia (changes in all four plasma lipid fractions: TC, HDL-C, TG, and LDL-C) [42]. In turn, cholesterol accumulation induces an inflammatory response, which is associated with the secretion of inflammatory cytokines [39]. Metabolic syndrome is closely related to the gut microbiota [93]. In addition to nutrition, numerous findings in human and animal models point to the critical contribution of sex hormones as major regulators of gut microbiota variability. In summary, subfertility can be seen as the “signature” of dysregulated homeostasis of the endocrine, immune, and metabolic pathways written in “GUT-ink”.

This study had some limitations. Firstly, this was a single-center study. Secondly, the number of patients may have been insufficient. A multi-center analysis with a larger patient base would be beneficial. Thirdly, this was a cross-sectional observational study and lacked a dynamic biochemical component. With this in mind, any cause-and-result relationships, although seemingly possible, should not be taken as definitive. Moreover, methodological limitations of our study include issues with research samples and selection, an insufficient sample size for statistical measurements, methods, and analyzers used to measure the concentrations of markers, limited access to data, and time constraints. This study did not contain clinical data/measurements, which could have supplemented the obtained results (such as clinical components of metabolic syndrome including waist circumference, waist-to-hip ratio, and blood pressure). In our work, we determined the concentration of markers that are used in the diagnosis of immunological, metabolic, hormonal, and nutritional changes. Therefore, we must have a broad view of this multilevel disorder. Additionally, there is a lack of previous studies on this multidisciplinary view of subfertility. Further longitudinal, large-scale, and multi-center studies are needed to confirm the results of this study.

## 5. Conclusions

An overlapping of lipid profile dysregulations, insulin resistance, thyroid autoimmunity, and type-2 inflammation was observed in subfertile individuals and couples diagnosed with unexplained subfertility. Meanwhile, healthy offspring need future parents to be in the best possible health condition. We propose one-time laboratory immune–metabolic diagnostics in both males and females. These may allow for a one-time assessment of targeted medical and nutritional interventions and help optimize patients’ health. Additionally, these results prompt the question: to what extent is unexplained subfertility a “syndrome of subfertility” consisting of decreased fertility and laboratory findings related to nutrition and gut health?

## Figures and Tables

**Table 1:** Biochemistries of the subfertile anti-TPO-positive compared with anti-TPO-negative female phenotype.

Parameter	TPO-Positive Subfertile Female Group (N = 14)	TPO-Negative Fertile Female Group (N = 23)	*p*-Value
anti-TPO Mdn (IQR) min–max	103.2 (22.5–233.5) 5.8–1000	0.150 (0.03–0.47) 0.0–1.3	<0.0001
anti-TG Mdn (IQR) min–max	38.4 (4.2–291.2) 0.7–563.6	1.6 (0.9–4.9) 0.56–49.8	0.002
TSH µIU/mL Mdn (IQR) 95% CI	2.34 (1.62–3.05) 1.79–2.91	1.29 (0.85–1.69) 1.12–1.73	0.003
fT4 ng/dL Mdn (IQR)	1.08 (1.02–1.23)	0.95 (0.89–1.02)	0.001
Insulin µU/mL Mdn (IQR)	8.3 (5.5–11.2)	5.2 (4.0–8.8)	0.060
HOMA-IR Mdn (IQR)	1.80 (1.08–2.61)	1.07 (0.84–1.80)	0.094
Sodium mmol/L Mdn (IQR)	139 (138–140)	140 (139–141)	0.049
Potassium mmol/L 95% CI	4.50 4.46–4.71	4.30 4.20–4.43	0.002

**Table 2:** Biochemistries of the subfertile low HDL-C cardio-metabolic female phenotype.

Parameter	_Low_HDL-C Subfertile Group (N = 14 Women)	_Optimal_HDL-C Fertile Group (N = 24 Women)	*p*-Value
HDL-C mg/dL Mdn (IQR)	44.00 (40.75–47.25)	62.50 (55.25–71.50)	<0.0001
Total Cholesterol /HDL-C Ratio	3.82 (3.46–4.70)	3.07 (2.62–3.42)	0.0002
Triglycerides /HDL Ratio	2.00 (1.73–2.84)	1.12 (0.79–1.37)	<0.0001
Triglycerides mg/dL	88 (79–116)	65 (55–89)	0.010
TSH µIU/mL Mdn (IQR)	1.33 (0.98–2.33)	1.29 (0.83–1.67)	0.661
Anti-TPO autoantibodies	0.36 (0.00–0.56)	0.19 (0.05–0.71)	0.880
Free T4 ng/dL Mdn (IQR)	1.08 (0.97–1.17)	0.95 (0.90–1.02)	0.018
Insulin µU/mL Mdn (IQR)	8.00 (5.25–11.15)	5.25 (4.05 –9.00)	0.063
HOMA-IR Mdn (IQR)	1.80 (1.07–2.52)	1.11 (0.86–2.00)	0.099
ALT	13.5 (8.0–20.0)	9.0 (7.0–12.8)	0.092
ALT > 19 IU/mL	5/14 (35.7%)	1/24 (4.2%)	0.019
AST/ALT	1.25 (1.00–1.84)	1.71 (1.5–1.96)	0.056
Sodium mmol/L Mdn (IQR)	139 (138–140)	140.0 (139–142)	0.019
Potassium mmol/L Mdn (IQR)	4.49 (4.28–4.85)	4.28 (4.13–4.44)	0.023
Total IgE	67.9 (22.2–99.7)	18.8 (11.7–27.4)	0.004
CRP	3.65 (1.33–9.03)	1.20 (0.70–2.30)	0.049
Fe	65.5 (46.5–82.5)	85.5 (47.3–106.5)	0.126
UIBC	239 (193–304)	212 (164–285)	0.169
IgA	1.8 (1.5–2.2)	1.9 (1.6–2.3)	0.628

**Table 3:** Results of the Spearman’s correlation between C-reactive protein (CRP) and metabolic indices in the study participants.

CRP vs.	Subjects Diagnosed with Subfertility		Fertile Subjects	
	R_S_	*p*-Value	R_S_	*p*-Value
Glucose	0.17	0.092	0.20	0.155
Insulin	0.39	<0.0001	0.25	0.082
HOMA-IR	0.40	<0.0001	0.29	0.041
Triglycerides	0.45	<0.0001	0.08	0.588
Cholesterol	0.16	0.116	−0.14	0.351
Ch/HDL-C	0.40	<0.0001	0.20	0.144
nonHDL-cholesterol	0.29	0.003	0.01	0.945
LDL-cholesterol	0.22	0.028	−0.01	0.979
HDL-cholesterol	−0.29	0.003	−0.25	0.078

Legend: R_S_—Spearman’s rank correlation coefficient; the strength of a correlation: R_S_ = 0.00 to 0.19: very weak correlation, R_S_ = 0.20 to 0.39: weak correlation; R_S_ = 0.40 to 0.69: moderate correlation.

**Table 4:** Biochemistries of the subfertile _High_HOMA-IR hepato-metabolic male phenotype.

Parameter	_High_HOMA-IR Subfertile Group (N = 13)	_Normal_HOMA-IR Fertile Group (N = 14)	*p*-Value
HDL-C mg/dL Mdn (IQR)	49 (45–54)	53 (47–65)	0.189
Total cholesterol/HDL-C Ratio	4.4 (4.2–5.5)	4.5 (3.6–4.8)	0.235
Triglycerides /HDL-C Ratio	2.7 (1.3–3.8)	1.7 (1.1–3.3)	0.216
Triglycerides mg/Dl	145 (70–186)	93 (67–148)	0.297
Total cholesterol mg/dL	227 (217–253)	231 (204–248)	0.560
LDL cholesterol mg/dL	162 151–175	147 132–164	0.090
TSH µIU/mL Mdn (IQR)	1.50 (0.91–1.79)	1.28 (0.92–1.64)	0.790
Free T4 ng/dL Mdn (IQR)	1.03 (0.98–1.09)	1.00 (0.94–1.11)	0.771
Glucose	91 (87–100)	87 (81–96)	0.080
Insulin µU/mL Mdn (IQR)	11.3 (9.6–15.9)	5.6 (4.9 –7.2)	<0.0001
HOMA-IR Mdn (IQR)	2.44 (2.08–3.82)	1.27 (1.08–1.46)	<0.0001
ALT IU/mL	32 (25–40)	15 (10–21)	0.0009
ALT > 30 IU/mL	8/13 (62%)	1/14 (7%)	<0.0001
AST/ALT	0.8 (0.7–1.1)	1.6 (1.0–1.9)	0.008
AST IU/mL (95% CI)	28 22–32 22.8–33.8	22 18–23 18.5–22.8	0.007
SHBG	30.0 (21.2–44.5)	43.7 (31.3–56.7)	0.049
Sodium mmol/L Mdn (IQR)	140 (139–141)	140 (139–142)	0.584
Potassium mmol/L Mdn (IQR)	4.49 (4.39–4.73)	4.46 (4.23–4.67)	0.680
Total IgE	29 (9–116)	47 (28–54)	0.827
CRP	1.2 (0.5–2.1)	1.3 (0.4–2.5)	0.865

**Table 5:** Biochemistries of the subfertile _High_IgE male phenotype.

Parameter	_High_IgE Subfertile Group (N = 13)	_normal_Serum IgE Fertile Group (N = 23)	*p*-Value
Total IgE kU/L	182 (136–271)	35 (9–54)	<0.0001
sIgE pollen sensitization N (%)	4/13 (30%)	1/23 (4%)	0.047
Non-atopic _high_IgE N (%)	9/13 (70%)	1/23 (4%)	<0.0001
Total cholesterol mg/dL	227.0 (193.0–233.5)	205.0 (179.0–236.0)	0.621
HDL-C mg/dL Mdn (IQR)	52.0 (40.5–62.5)	51.0 (42.0–60.0)	0.961
Triglyceride mg/dL Mdn (IQR)	116.0 (64.0–134.5)	103.0 (76.0–145.0)	0.987
TSH µIU/mL Mdn (IQR)	1.35 (0.87–1.56)	1.21 (0.79–1.81)	1.00
Anti-TPO autoantibodies	0.31 (0.19–0.69)	0.14 (0.05–0.30)	0.0097
Free T4 ng/dL Mdn (IQR)	1.05 (1.03–1.9)	1.00 (0.93–1.04)	0.007
Sodium mmol/L Mdn (IQR)	140 (139–141)	141.0 (139–142)	0.086
Potassium mmol/L Mdn (IQR)	4.48 (4.38–4.71)	4.43 (4.18–4.66)	0.347

**Table 6:** (**A**) Predictors of male subfertility included in the multivariate binary logistic regression (indicating two male endophenotypes: _High_HOMA-IR and _High_IgE). (**B**) Predictors of female subfertility included in the multivariate binary logistic regression (indicating two female endophenotypes: _Low_HDL and _High_aTPO). (**C**) Predictors of couple (male and female) subfertility included in the multivariate logistic regression.

A				
Predictor	Odds Ratio	Std Error	*p*-Value	95% Confidence Interval
HOMA-IR > 1.9 vs. <1.9 (_High_HOMA-IR)	6.07	4.9	0.025	1.25; 29.50
Total IgE > 100 vs. <100 kU/L (_High_IgE)	6.37	5.5	0.034	1.15; 35.11
Prediabetes: Yes vs. No	2.7	3.3	0.407	0.25; 28.59
HDL-C < 40 vs. >40 mg/dL	0.56	0.57	0.571	0.078; 4.08
Anti-TPO antibodies: Yes, No	5.70	6.9	0.152	0.53; 62.17
Anti-TG antibodies: Yes, No	0.55	0.51	0.523	0.089; 3.4
Cons	1.37	0.063	0.700	0.056; 3.36
B				
Predictor	Odds Ratio	Std Error	*p*-value	95% Confidence Interval
HDL-C < 50 mg/dL (_Low_HDL)	10.92	11.95	0.029	1.27; 93.27
Anti-TPO positivity (_high_aTPO)	5.48	4.70	0.047	1.02; 29.40
Anti-TG positivity	1.12	0.65	0.840	0.36; 3.47
Hyperinsulinemia > 12 µU/mL	1.92	2.34	0.594	0.18; 21.03
Total IgE > 100 kU/L	4.87	5.54	0.164	0.52; 45.25
Cons	0.87	0.33	0.727	0.41; 1.86
C				
Predictor	Odds Ratio	Std Error	*p*-value	95% Confidence Interval
Total IgE > 100 kU/L	6.50	5.56	0.029	1.22; 34.74
No. of laboratory components of MetS *	1.68	0.43	0.046	1.01; 2.78
Anti-TPO positivity	4.22	3.67	0.098	0.76; 23.25
Single sign of lipid profile dysregulation **	0.23	0.17	0.046	0.05; 0.98
Cons	1.14	0.52	0.779	0.46; 2.78

* Analyzed components of metabolic syndrome, insulin resistance, and dyslipidemia: fasting serum glucose FSG > 100 mg/dL, fasting serum insulin FSI > 12 µU/mL, HOMA-IR > 1.9, HDL < LRL, TG > 150 mg/dL, TC/HDL-C > 4. ** At least one of the three components of the lipid profile: HDL < LRL, TG > 150 mg/dL or TC/HDL-C > 4.

## Data Availability

All data analyzed during this study are available within this paper.

## Supplementary Materials

The following supporting information can be downloaded at: https://www.mdpi.com/article/10.3390/jcm12186094/s1, Figure S1: Distribution of laboratory components of metabolic syndrome in subfertile and fertile couples. In 20 out of 50 (40%) subfertile couples, components of MetS were found both in the male and female as compared with 3 out of 25 fertile couples (12%) (*p* = 0.017; Fisher’s exact test; two-sided *p*-value); Table S1: Characteristics of thyroid function and thyroid autoimmunity markers in the study participants; Table S2: Characteristics of lipid profiles in the study subjects; Table S3: Characteristics of laboratory measurements in the subfertile and fertile study groups.
