# Supplementary material for: Subfertility as Overlapping of Nutritional, Endocrine, Immune, and Cardiometabolic Dysregulations—A Study Focused on Biochemical Endophenotypes of Subfertile Couples
Source: J Clin Med. 2023 Sep 21;12(18):6094. doi: 10.3390/jcm12186094 (PMC10531916; doi:10.3390/jcm12186094)

Supplementary

Table S1 Characteristics of thyroid function and thyroid autoimmunity markers in the study participants.

	Participants diagnosed with subfertility	Fertile subjects with at least 3 children	p-value
N/ Male	100 / 50	50 / 25	
TSH (µIU/mL) (IQR)	1.38 (0.94-1.81)	1.29 (0.82-1.72)	0.399[U]
TSH <1.5 (%)	58	58	0.964[F]
TSH 1.5 < 2.5 (%)	33	34	
TH≥ 2.5 (%)	9	8	
Female	1.44 (1.00-1.96)	1.29 (0.84-1.68)	0.315[U]
Male	1.29 (0.92-1.65)	1.21 (0.80-1.80)	0.783 [U]
Free T4 (ng/dL) (IQR)	1.10 (1.02-1.19)	0.96 (0.92-1.02)	<0.0001[U]
% of Reference Range	51.3%	33.3%	
Female	1.13 (1.03-1.20)	0.95 (0.89-1.02)	<0.0001[U]
% of Reference Range	88.6%	32.1%	
Male	1.08 (1.02-1.16)	1.00 (0.93-1.07)	0.0015[U]
% of Reference Range	48.7%	38.5%	
Anti-thyroid peroxidase antibodies (anti-TPO) (IU/mL)			
Q2 (IQR)	0.44 (0.14-1.41)	0.15 (0.05-0.33)	0.003[U]
90th Percentile	112.0	1.2	
Positive anti-TPO, N (%)	22 (22%)	4 (8%)	0.039[F]
TPO-positive sub-group (IQR)	98.0 (37.4-256.0)	232 (7.4-322.6)	0.644[U]
Female			
Q2 (IQR)	0.52(0.20-12.05)	0.17 (0.04-0.69)	0.028[U]
Positive anti-TPO, N (%)	14 (28%)	2 (%)	0.071[F]
Male			
Q2 (IQR)	0.32(0.11-0.74)	0.12 (0.05-0.27)	0.027[U]
Positive anti-TPO, N (%)	8 (16%)	2 (8%)	0.480[F]
Anti- thyroglobulin antibodies (anti-TG) (IU/mL)			
Q2 (IQR)	2.17 (0.90-8.76)	1.20 (0.87-4.70)	0.105[U]
Positive anti-TG, N (%)	34 (34%)	15 (30%)	0.713 [F]
TG-positive sub-group (IQR)	30.6 (7.8-105.5)	8.5 (4.9-49.0)	0.074[U]
Female			
Q2 (IQR	3.35 (1.08-26.18)	2.99 (0.92-5.66)	0.234[U]
90th Percentile	239.7	28.6	
Positive anti-TG, N (%)	24 (48%)	10 (40%)	0.625 [F]
Male			
Q2 (IQR	1.55 (0.83-3.93)	0.95 (0.78-2.48)	0.158[U]
90th Percentile	30.79	61.15	
Positive anti-TG, N (%)	10/50 (20%)	5/25 (20%)	1.000[F]

[F] = Fisher's exact test; two-sided P; [U] = Mann-Whitney U test, two-tailed P; Q2-median (Q2-Mdn), IQR - interquartile range; Levels of anti-TG > 4.110 IU/mL and anti-TPO > 5.610 IU/mL were defined as antibody positive.

Table S2. Characteristics of lipid profiles in the studied subjects.

	Subjects diagnosed with subfertility	Fertile couples with at least 3 children	p-value
N/ Male	100 / 50	50 / 25	
Total Cholesterol (mg/dL) (IQR)	198 (169-218)	195(180-228)	0.346^{U}
Female	179 (164-206)	186 (176-218)	0.185^{U}
Male	213 (178-229)	206 (183-234)	0.866^{U}
Non-HDL-C (mg/dL)	137 (111-166)	139 (117-164)	0.735^{U}
Female	122 (108-143)	124 (108-153)	0.700^{U}
Male	155 (127-178)	147 (132-180)	0.870^{U}
LDL-C (mg/dL)	116 (96-142)	120 (106-144)	0.428^{U}
Female	106 (91-128)	110 (99-134)	0.283^{U}
Male	133 (108-157)	127 (113-157)	0.928^{U}
LDL-C>130 mg/dL, N (%)	43 (43%)	21 (42%)	1.000^{F}
TC to HDL-C Ratio	3.46 (2.99-4.38)	3.45 (2.90-4.20)	0.708^{U}
Female	3.13 (2.85-3.72)	3.15 (2.64-3.45)	0.312 ^{U}
Male	3.95 (3.24-4.65)	4.13 (3.41-4.56)	0.698^{U}
TC/HDL>4, Male, N (%)	26 (52%)	14 (56%)	0.809^{F}
HDL-C (mg/dL)	54 (46-63)	56 (51-64)	0.222^{U}
Female	55.5 (48.8-64.0)	62 (54.5-71.0)	0.032^{U}
Male	52.5 (44.8-59.5)	51 (42.5-58.5)	0.723^{U}
HDL-C <50 mg/dL (Female) (%)	14 (28%)	1 (4%)	0.015^{F}
HDL-C <40 mg/dL (Male) (%)	4 (8%)	3 (12%)	0.680^{F}
Triglycerides (mg/dL)	82 (58-116)	87 (62-117)	0.458^{U}
Female	66 (56-93)	66 (57-93)	0.995^{U}
Male	100 (66-128)	103 (80-156)	0.271^{U}
TG to HDL-C Ratio	3.5 (3.0-4.4)	3.5 (2.9-4.2)	0.706^{U}
Female	1.2 (0.9-1.9)	1.1 (0.8-1.4)	0.438^{U}
Male	1.8 (1.2-3.2)	2.2(1.2-3.4)	0.266^{U}
Male Subgroup with LDL-C >120 mg/dL:			
N (%)	31 (62%)	16 (64%)	1.000 ^{F}
HOMA-IR	1.82 (1.24-2.29)	1.33 (1.11-1.73)	0.049^{U}
HOMA-IR>1.9	13 (42%)	2 (13%)	<0.001^{F}
ALT (IU/mL)	24 (16-32)	15 (10-24)	0.016^{U}
AST to ALT Ratio	0.90 (0.74-1.31)	1.40 (0.92-1.87)	0.039^{U}

^{F} = Fisher's exact test; two-sided P; ^{U} = Mann-Whitney U test, two-tailed P; Q2- Mdn, IQR - interquartile range

Table S3. Characteristics of laboratory measurements in the subfertile and fertile study groups.

	Participants diagnosed with subfertility	Fertile subjects with at least 3 children	p-value
N/ Male	100 / 50	50 / 25	
Total IgE (kU/L) (IQR)	32.8 (15.9-84.1)	24.8 (11.8-45.8)	
90th Percentile	180.7	82.4	0.016[U]
Total IgE>100 kU/L N (%)	21 (21%)	3 (6%)	0.019[F]
Specific-IgE sensitization N (%)	13 (13%)	3 (6%)	0.265[F]
IgA (g/L) (IQR)	2.2 (1.6-2.6)	1.9 (1.7-2.5)	0.349[U]
Female	2.1 (1.6-2.5)	1.9 (1.5-2.3)	0.317[U]
Male	2.2 (1.7-3.0)	1.9 (1.8-2.9)	0.609[U]
SHBG (nmol/L) (IQR)	49.9 (31.9-70.6)	49.6 (36.4-69.4)	0.811[U]
Female	65.8 (52.1-90.6)	66.0 (49.2-87.9)	0.937[U]
Male	37.2 (26.5-49.4)	39.0 (31.2-49.6)	0.455[U]
ALT IU/mL	14 (9-24)	13 (8-18)	0.140[U]
ALT>19 IU (Female) N (%)	9 (18%)	1 (4%)	0.150[F]
ALT>30 IU (Male) N (%)	14 (28%)	3 (12%)	0.151[F]
ASP IU/L	18 (16-23)	18.5 (15.5-22)	0.903[U]
CRP mg/L	1.1 (0.3-2.1)	1.4 (0.6-2.3)	0.124[U]
Creatinine mg/dL	0.78 (0.71-0.91)	0.80 (0.75-0.90)	0.563[U]
Fe µg/dL	93 (71-127)	94.5 (69.8-124)	0.936[U]
UIBC µg/dL	198 (165-248)	207 (161-235)	0.507[U]
Na mmol/L (IQR)	139 (138-140)	141 (139-142)	0.0005[U]
Female	139.0 (138.0-140.0)	140.0 (139.0-141.5)	0.006 [U]
Male	140.0 (139.0-141.0)	141.0 (139-142.5)	0.020 [U]
K mmol/L (mean ±SEM)	4.46 ±0.03	4.38 ±0.04	0.099[U]
Female, mean ±SEM	4.48±0.03	4.29 ±0.04	0.023 [U]
Male, mean ±SEM	4.47 ±0.05	4.45 ±0.05	0.995[U]
Fasting glucose (mg/dL) (Mdn) (IQR)	87.0 (83.0-94.8)	87.0 (82.8-93.0)	0.652[U]
Female	86.0 (83.0-93.0)	86.0 (83.5-91.5)	0.835[U]
Male	89.5 (83.8-96.3)	87.0 (83.5-95.5)	0.605[U]
Hyperglycemia (≥ 100 mg/dL), N (%)	9 (9%)	4 (8%)	1.000[F]
Female	3 (6%)	3 (12%)	0.394[F]
Male	6 (12)	1(4%)	1.000[F]
Fasting Insulin (uU/mL) (Mdn) (IQR)	6.5 (4.8-9.8)	5.6 (4.7-8.4)	0.122[U]
Female	6.2 (4.7-9.5)	5.3 (4.1-10.0)	0.298[U]
Male	8.3 (4.9-10.1)	5.7 (5.2-7.9)	0.216[U]
Hyperinsulinemia (>12 uU/mL), N(%)	13 (13%)	1 (2%)	0.036[F]
Female N (%)	5 (10%)	1 (4%)	0.657 [F]
Male N (%)	8 (16%)	0 (0%)	0.046 [F]
HOMA-IR (Mdn) (IQR)	1.47 (0.96-2.06)	1.28 (1.01-1.80)	0.170[U]
Female	1.34 (0.92-2.02)	1.15 (0.88-1.96)	0.455[U]
Male	1.53 (1.10-2.22)	1.43 (1.12-1.73)	0.192[U]

[F] = Fisher's exact test; two-sided P; [U] = Mann-Whitney U test, two-tailed P; IQR - interquartile range; SEM - standard error of mean.

Figure S1. Distribution of laboratory components of metabolic syndrome in subfertile and fertile couples.

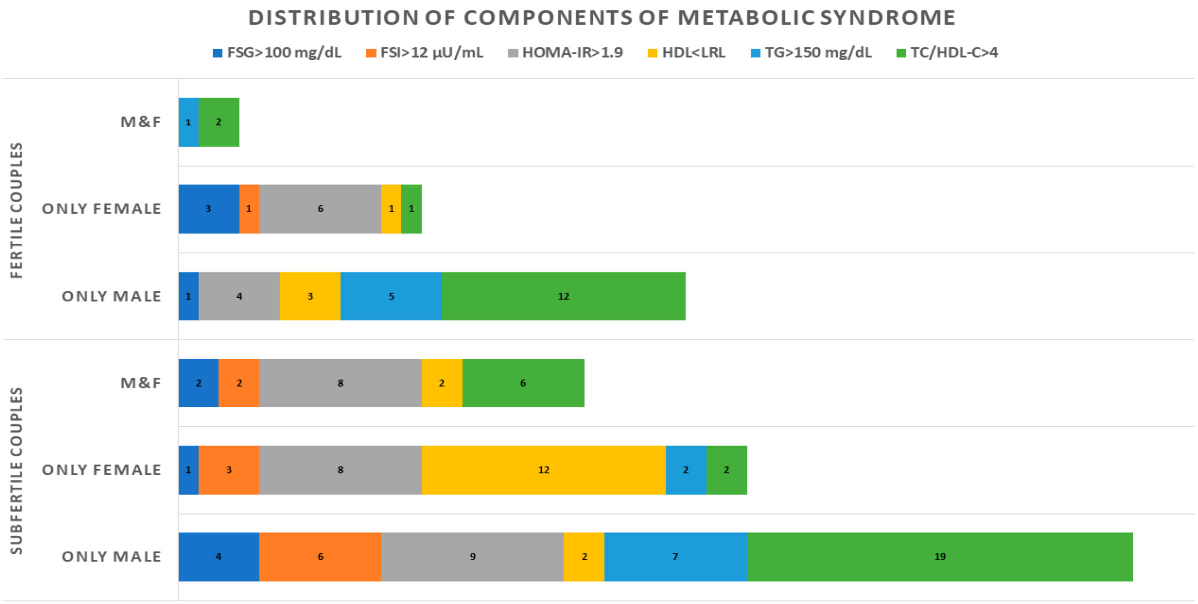

Supplement: Supplementary file 1 [file jcm-12-06094-s001.zip › jcm-2552193-supplementary.pdf]
